# Assessing the Association of Food Preferences and Self-Reported Psychological Well-Being among Middle-Aged and Older Adults in Contemporary China-Results from the China Health and Nutrition Survey

**DOI:** 10.3390/ijerph15030463

**Published:** 2018-03-07

**Authors:** Yen-Han Lee, Mack Shelley, Ching-Ti Liu, Yen-Chang Chang

**Affiliations:** 1 Department of Applied Health Sciences, School of Public Health, Indiana University, Bloomington, IN 47405, USA; 2 Department of Political Science, Department of Statistics, Iowa State University, Ames, IA 50011, USA; mshelley@iastate.edu; 3 Department of Biostatistics, School of Public Health, Boston University, Boston, MA 02118, USA; ctliu@bu.edu; 4 Center for General Education, National Tsing Hua University, Hsinchu City 300, Taiwan

**Keywords:** food preferences, psychological well-being, China, aging, dietary behaviors

## Abstract

China has undergone rapid social transitions within the last few decades. However, mental health issues, challenges to psychological well-being, and poor dietary choices have gradually surfaced. These health concerns are related to the rapid growth of the aging population and of the fast-paced industrialized society. Nevertheless, there is little knowledge about food preferences and psychological well-being measurements in contemporary China. Applying the 2011 China Health and Nutrition Survey (CHNS) with a cross-sectional study design (*n* = 7970), we conducted multinomial logistic regression models to investigate the associations of food preferences, including fast food, salty snacks, fruits, vegetables, and sweetened beverages, with psychological well-being among Chinese middle-aged and older adults (age ≥ 45). Food preferences are mostly associated with psychological well-being (*p* < 0.05). However, respondents’ preferences regarding fast food, salty snacks, and sweetened beverages are associated not only with poorer psychological health status, but also with positive psychological well-being. We speculate that Chinese older adults may consume Westernized fast food and salty snacks as pleasure to the palate due to the recent Westernization in modern China. We also provide practical implications of results from this preliminary study.

## 1. Introduction

### 1.1. Background

China has experienced dramatic Westernization and industrialization since the early 21st century. These rapid social reforms have increased China’s role globally, including its diplomatic presence, global governance, economy, and cultural presence [1]. However, the contemporary pattern of Westernization comes with challenges, such as public health. For example, the prevalence of non-communicable diseases (NCDs) has become the leading cause of death in China and most NCDs are associated with Western lifestyle and dietary patterns [2]. Among 10 million deaths caused by different diseases each year, NCDs account for more than 80% [2,3]. Additionally, China’s fast-paced industrialized society has led to residents’ mental health issues [4], making mental disorders one of the most concerning public health threats in contemporary China.

China and India account for one-third of the global burden of mental health diseases, including depressive disorders, anxiety, attention deficit hyperactivity disorder (ADHD), drug use disorders, and others [5]. Furthermore, the mental health situation is exacerbated in both China and India because their populations continue to age rapidly [5]. Without effective health promotion programs, the growing mental health issues and the rapidly growing aging population will continue to threaten people’s overall health. It is critical to assess the detailed local context at provincial or district levels of mental health service provision [6].

Psychological well-being is related to many positive aspects, including cognitive function, social relationships, and health [7]. As defined by Ryff and Keyes [8], the structure of psychological well-being has six primary factors: self-acceptance, personal growth, purpose in life, environmental mastery, autonomy, and positive relations with others. These factors explain that people with distinct background (i.e., personal experience and living environment) or socioeconomic status might have different psychological well-being. Individuals might experience mood shifts under different social settings. Thus, it becomes important for Chinese individuals to adjust their lifestyles and attitudes to obtain better psychological well-being after dramatic Westernization.

With the hope of reducing mental health problems, the Chinese central government has established plans to promote mental health knowledge, reduce stigma, increase financial support for mental services, and develop psychological well-being services [9]. One of the latest developments in China is the provision of psychological well-being services, which aims to emphasize the importance of other aspects of mental health issues besides psychiatry [9]. This psychological well-being management plan aims to help individuals with mental problems, but it also supports the general population with specific needs [9]. However, research related to psychological well-being remains scarce in China.

### 1.2. Purpose of the Study

Food intake and psychological well-being have been assessed in a few studies [10,11]. In the qualitative study by von Essen and Martensson [10], food intake is a necessity of life and food is helpful for relieving uncomfortable memories. Another study [11] found that better psychological well-being is associated with higher consumption of fresh vegetables and fruits in Lithuanian adults aged 45 to 72 years. In addition, social and emotional influences are strongly associated with eating behaviors and food choices [12,13]. Research conducted in Northwestern England has demonstrated that childhood experience and adult well-being are associated with adult eating behaviors (18 to 95 years) and food preferences [14]. Furthermore, an organic food lifestyle gives young adults a positive health perception (18 to 33 years old) and psychological stability [15], as organic dietary patterns connect with young adults’ identity and well-being on a psychological level [15].

To date, no research has investigated the association between food preferences and psychological well-being in China. Such research is needed, as China has faced dramatic social transitions, Westernization, changes in dietary patterns, and an aging population. Moreover, it is also believed that the growing aging population and heightened incidence of mental health go hand-in-hand [5]. In fact, Westernized food options, such as fast food, have grown steadily in China. Fast food establishments grew from 929,125 to 1,981,019 units and revenues skyrocketed from $25,694 million to $87,239 million between 2004 and 2012 [16]. In the eleven-year span from 1990 to 2001, the consumption of grain and vegetables showed dramatic declines, from 289 to 176 pounds per person and 306 to 256 pounds per person, respectively [17]. These indicators have demonstrated the rapid shifts of the dietary landscape in China.

With the rapid social transition, there is a concrete need to fill this literature gap regarding food preferences and psychological well-being, given that studies have shown strong associations in the Western world [11,14]. China, a country with rapid Westernization, could face similar health issues in the near future. For example, the Chinese traditional food options include healthy cooking styles, such as boiling and steaming, but the current options also include increasing trends of frying food and rapid growth of the modern food system [18], largely due to this rapid Westernization. Therefore, it is important to address what types of food preferences could challenge Chinese residents’ psychological well-being.

## 2. Materials and Methods

### 2.1. Study Sample: China Health and Nutrition Survey

The China Health and Nutrition Survey (CHNS) is a longitudinal survey and open cohort conducted from 1989 to the currently available 2011 wave, with multistage and random cluster procedures. This comprehensive dataset aims to study the influences of nutrition, health, and family planning policies established by both national and local government agencies in China. Furthermore, the CHNS investigates the impact of social and economic transitions in Chinese society on residents’ overall health and nutrition status. Nine provinces were surveyed: Liaoning, Heilongjiang, Jiangsu, Shandong, Henan, Hubei, Hunan, Guangxi, and Guizhou. An additional three mega-cities (municipals)—Beijing, Chongqing, and Shanghai—were added in the latest survey. In total, 5884 households from 42 towns and 126 villages were included in the 2011 wave [18]. Popkin et al. [19] and Zhang et al. [18] provide further information for the CHNS.

For this research we focused on the associations between food preferences and psychological well-being among middle-aged and older adults who were at least 45 years old (age ≥ 45). This selection was applied because psychological well-being measurements were specifically designed for these respondents, according to the CHNS. The original study sample included 9551 respondents. We excluded participants who did not fully answer questions of interest for this present study and removed missing values. With our selection and exclusion, the final study sample includes 7970 individuals who answered all questions of interest.

We used de-identified and publicly-available datasets from the official CHNS website. Hence, approvals from Institutional Review Boards were not required at authors’ institutions.

### 2.2. Outcome Variables

We selected three categorical outcome variables as self-reported psychological well-being measurements from the 2011 CHNS questionnaire: 1. “I had as much pep as I had in 2010” (“Pep” refers to “energy” in American English); 2. “I am as happy now as when I was younger”; 3. “As I get older, things are better than I thought they would be.” According to the questionnaire, the coding included “neutral”, “agree”, and “disagree”. The neutral response, neither agree nor disagree, was treated as the reference category in our analysis. All descriptions of variables were translated from Mandarin Chinese to English by the CHNS investigators and slightly edited by the authors of this research. 

### 2.3. Primary Predictors and Covariates

The primary predictor of interest is food preferences based on responses to five food classification questions. These food options include fast food (such as pizza and hamburgers), salty snacks (such as pretzels, potato chips, and French fries), fruits, vegetables, and sweetened beverages (including both soft drinks and sweetened fruit drinks). Each food preference was coded with one of these potential responses: dislike, neutral (neither dislike nor like), like, and do not eat this type of food. The neutral preference was treated as the reference category in all statistical analyses.

We selected our covariates based on the theoretical framework of micro- and macro-environments, reframed by Swinburn and Egger [20]. The micro-environments were personal factors (e.g., age, gender, and level of education) while macro-environments were determinants at a higher level (e.g., province and community). The following variables were employed as covariates in all statistical models: age (a continuous variable), gender (male or female, a dichotomous variable), disease status (no or yes, a dichotomous variable), employment status (no or yes, a dichotomous variable), marital status (no or yes, a dichotomous variable), educational attainment (lower education, middle/high school education, technical school, university and above; a categorical variable), gross household income quintile (1–5, a categorical variable), community (rural or urban, dichotomous variable), and province (Northeast, East Coastal, Central, South, municipals; a categorical variable).

Age and gender were selected as basic biological information. Disease status was considered to determine whether respondents had either chronic or acute disease within four weeks before they completed the survey. Disease status was selected because previous studies have observed associations between chronic diseases and psychological well-being, including multiple sclerosis [21] and coronary heart disease [22]. Employment status (retirement age in China: for men is 60 years old, and for women between 50 and 55 years old), marital status, educational attainment, and gross household income quintiles were used to describe individuals’ basic socioeconomic status information. For the variable of gross household income quintiles, the first quintile represents respondents with the lowest gross household income, while the fifth quintile represents respondents with the highest gross household income. In the CHNS survey, the gross household income was calculated in Renminbi (RMB), the Chinese currency. Regional information includes both community and provincial variables. For the five categories of the provincial variable, northeast includes Liaoning and Heilongjiang; Jiangsu and Shandong are under east coastal; central provinces are Hunan and Hebei; southern provinces are Hunan, Guangxi, and Guizhou; and municipals include Beijing, Chongqing, and Shanghai.

### 2.4. Statistical Analyses and Multinomial Logistic Regression

We applied multinomial logistic regression models for this cross-sectional research. Multinomial regression is a rigorous modeling technique when outcome variables have more than two categories [23]. Results are reported for all statistical analyses (relative risk ratio (RRR), beta coefficients (β), and standard errors (SE)). Further, we performed the Benjamini-Hochberg procedure (FDR) to adjust calculated *p*-values, a commonly-used and practical method to control the false discovery rate [24]. Prevalence rates of each food preference on psychological well-being also were calculated. All statistical analyses were conducted by the free and publicly available statistical package R (version 3.3.3) using its package “nnet” for multinomial logistic regression. We used the R package “gmodel” to calculate prevalence rates on each food preference with measures of psychological well-being.

## 3. Results

### 3.1. Characteristics of the Study Sample

We analyzed a total of 7970 individuals whose mean age was 60 years old (*SD* = 10). Among them, the majority were female (52.3%), married (85.9%), unemployed (53.2%), and resided in rural areas (57.4%). Most individuals were healthy without either chronic or acute disease. On the other hand, only a small number of respondents had technical education or above and most respondents had gross household income below the third quintile (Table 1). In general, most respondents agreed with each psychological well-being statement and had healthier food preference knowledge (Table 1). Table 1 provides comprehensive characteristics for the study sample.

### 3.2. Prevalence Rate of Food Preferences and Psychological Well-Being

People who disliked fast food and salty snacks had a higher probability of agreeing that they were as energetic as in the previous year. For study subjects who liked vegetables and fruits, the proportions who agreed they are as energetic as in the previous year were larger than those who disliked vegetables and fruits (Table 2). The proportion of individuals who disliked sweetened beverages agreeing that they are as energetic as in the previous year was larger than for those who liked sweetened beverages. In the same vein, respondents who had healthier food preferences were more likely to report better psychological well-being (Table 2). Table 2 provides comprehensive prevalence rates on food preferences with measures of psychological well-being.

### 3.3. Associations of Food Preferences with Psychological Well-Being

For “I had as much as pep as I had in 2010” (Model 1), individuals who liked fast food had higher chances of agreeing with the statement (RRR = 2.11, *p* < 0.01), and respondents who disliked, liked, and did not eat fast food had higher chances of disagreeing with the statement, compared to those who had a neutral attitude (Table 3 and Table 4). Individuals who liked salty snacks had higher chances of agreeing that they had the same energetic level as last year (RRR = 1.65, *p* < 0.05). Participants who liked or disliked sweetened beverages had higher chances of either agreeing or disagreeing that they have the same energetic level (*p* < 0.01), compared with those who had a neutral attitude. Respondents who disliked fruits had higher chances of disagreeing with the statement, and those who liked fruits had higher chances of agreeing (Table 3 and Table 4). Individuals who like vegetables had higher chances of agreeing that they had the same pep as in 2010 (Table 3 and Table 4).

In the second model of “I am as happy now as when I was younger”, respondents who disliked fast food had higher chances of agreeing that they were as happy as in their younger years (RRR = 1.43, *p* < 0.01). Individuals who liked fast food had higher chances of disagreeing with the statement (Table 3 and Table 4). Respondents who did not eat fast food had higher chances of disagreeing with the statement (RRR = 1.87, *p* < 0.01). The chances of agreeing and disagreeing with the statement were all higher among respondents who liked and disliked fruits, compared with those who had a neutral attitude; the observations for sweetened beverages are also in the same vein (Table 3 and Table 4). Furthermore, respondents who like vegetables had higher chances of agreeing with the statement (RRR = 1.70, *p* < 0.01).

In the third model, respondents who liked, disliked, and did not eat fast food had higher chances of agreeing with the statement “As I get older, things are better than I thought they would be” (Table 3 and Table 4). In terms of preferences on fruits and sweetened beverages, respondents who liked and disliked these two food options all had higher chances of agreeing with the statement, compared to those who had a neutral attitude. Respondents who like vegetables had higher chances of agreeing that they have better overall feelings (RRR = 1.81, *p* < 0.01). Respondents who prefer sweetened beverages also had higher chances of disagreeing with the statement, compared with those who had a neutral attitude (Table 3 and Table 4). Nevertheless, participants who liked or disliked sweetened beverages all had higher chances to agree with the statement. 

## 4. Discussion

This preliminary study explored the associations of food preferences with psychological well-being among middle-aged adults and elders in contemporary China. In general (Table 3 and Table 4), fast food, fruits, vegetables, and sweetened beverages are associated with all outcome variables, except between salty snacks and the measurement of “As I get older, things are better than I thought they would be” (Model 3). We also observed some remarkable results. First, Chinese older adults’ preferences for fast food, salty snacks, and sweetened beverage consumption are not always associated with higher chances of reporting poorer psychological well-being in all statistical models. Indeed, older adults who liked salty snacks had higher chances of reporting better energy and happiness in the present study. This is inconsistent with previous findings that consumption of fast food and commercially baked goods are associated with higher risks of depression [25]. In the same vein, Zahedi et al. [26] also observed that junk food, such as sweetened beverages, fast food, and salty snacks, increases the risk of psychiatric distress and violent behaviors among Iranian children and adolescents.

Contrary to the previous findings of Sanchez-Villegas et al. [25] and Zahedi et al. [26], one potential explanation for our observations is that China has experienced rapid Westernization only within the last few decades. For example, “Kentucky Fried Chicken” (KFC)—the famous fast food chain restaurant from the United States—did not establish its first branch in China until 1987 [27]. Given that our focus is on Chinese older adults, we speculated that the concept of Westernized fast food remains novel to those respondents. Furthermore, various food options may actually increase happiness among consumers [28]. Chinese older adults may try Westernized food options, including fast food and salty snacks, as new pleasures. However, not only could fast food consumption be associated with poorer psychological condition [25,26]; fast food is also linked with obesity and overweight among Chinese adolescents [29]. This existing knowledge gap suggests that the association between fast food consumption and psychological well-being, and the potential gap between younger and older adults, should be studied further. As Chinese Westernization did not take place until the last few decades, it is possible that older Chinese adults maintain traditional dietary patterns. For instance, in Chinese society, the traditional dietary pattern of “morning tea” (i.e., Cantonese breakfast) is regarded as strengthening friendships and social activities [30]. This type of traditional setting is common in China. The taste of Westernized food might serve only as a personal pleasure. On the other hand, older Chinese adults may not be aware of Westernized food options on the table, which may be another explanation why some results in our research are not consistent with previous studies.

Sweetened beverages are another concern in China. In just the ten-year span from 2000 to 2010, the daily per capita sales of Pepsi and Coca-Cola increased by 127% and 145%, respectively [31]. Not only does liking sweetened beverages increase the risk of NCDs, such as diabetes [32], but sweetened beverage intake is also strongly associated with Chinese adults’ smoking behavior; frequent sweetened beverage consumers are more likely to smoke [33]. However, similar to the findings regarding fast food and salty snacks, we observed that sweetened beverage preference among older Chinese adults does not always disagree with psychological well-being statements. The sweetened beverage industry has linked its products with happiness and family relationships. In fact, many sweetened beverage companies, such as PepsiCo and Coca-Cola, market their products with acceptable social aspects and neglect to present the associated health risks [34]. Under such circumstances, individuals who liked sweetened beverages may not be aware of the harmful effects on human health. We suggest that public health practitioners and researchers should continue to focus on the psychological effects of sweetened beverages on human beings in China, including older adults. Public health promotion programs should play a key role in investigating this consumption behavior in the long term.

Compared with the findings from fast food, salty snacks, and sweetened beverages, the associations that involve liking vegetables are not as surprising (Table 3 and Table 4). Russell et al. [14] observed that individuals who had adverse childhood experiences had a higher probability of lower consumption of vegetable and fruit portions; lower vegetable and fruit consumption is associated with greater health risks. Mujcic and Oswald [35] also pointed out that greater fruits and vegetables consumption can increase happiness, life satisfaction, and well-being. However, our results regarding preferences for fruits are more complicated than those for vegetables. Sharma et al. [36] suggested that fruit consumption can help reduce obesity, and that different fruit intake mechanisms (such as 100% fruit juice) could lead to obesity given that most fruits contain some sugar. Since the CHNS does not have measurements for different types of fruits, we need to be very cautious in claiming direct associations between fruit preferences and psychological well-being, although we did find strong associations. Other measurements regarding various types of fruit intake or long term consumption should be considered in further research.

China has faced public health issues through industrialization and Westernization in the 21st century [37]. With the hope of promoting healthy lifestyles and reducing NCDs, the Chinese central government has announced plans to enhance health promotion strategies, including “Healthy China 2030” [37]. Variables related to poor health lifestyle are also associated with poor dietary options [2,18]. With the trend of increasing mental health issues in China [4,5], the Chinese central government should also take further actions to prevent problems with mental health and improve overall psychological well-being among older adults because China’s rapid growth of its aging population is another public health challenge.

## 5. Conclusions

Since this is a preliminary assessment, we should also point out several limitations and further research directions. First, we applied a cross-sectional research design to investigate associations so we cannot establish causality in the present study. Second, we used food preferences to determine individuals’ dietary patterns. Other research should be conducted on servings of each food item among Chinese residents to capture dietary patterns especially for Western fast food (i.e., serving portions). Although the CHNS database includes the three-day 24-h dietary recall method for some types of food, this method was unable to capture respondents’ long-term dietary behaviors, which could impact residents’ mental health. To further understand the association between food consumption and psychological well-being, comprehensive self-reported intake frequency of food consumption should be considered. Additionally, because the CHNS survey asked questions related to psychological well-being only to older respondents (45 years old and above), we do not have information to investigate younger individuals (44 years old and below). Researchers should continue to look into similar topics of interest and compare the differences in trends between older and younger adults using other comprehensive datasets. Finally, because only limited measurements on psychological well-being are available in the CHNS, further research should be conducted with comprehensive related questions.

Despite these limitations, this preliminary study provides valuable lessons for discussing the associations of food preferences with psychological well-being among Chinese middle-aged and older adults. We have found that preferences of fast food, salty snacks, and sweetened beverages are not always associated with poorer psychological well-being. As such, there is a high chance that the Westernized food preferences remain novel to older respondents in this research. More research is urgently needed to investigate this topic of interest. Thus, it is important for public health practitioners to understand what the most effective dietary patterns are to improve older adults’ psychological well-being. Additionally, further research should continue to focus on investigating the impact of such concerns on mental health and psychological well-being in China, especially among Chinese middle-aged individuals and elders and the public health concerns associated with Westernized lifestyles.

## Figures and Tables

**Table 1:** Frequency distributions for the final study sample (*n* = 7970). Data: China Health and Nutrition Survey, 2011.

Variables	Measurements	Observations (*n*, %) (*n* = 7970)
*Psychological well beings:*		
“I had as much pep as I had in 2010”	Neutral	2086 (26.2)
	Disagree	2400 (30.1)
	Agree	3484 (43.7)
“I am as happy now as when I was younger”	Neutral	2460 (30.9)
	Disagree	2053 (25.8)
	Agree	3457 (43.4)
“As I get older, things are better than I thought they would be”	Neutral	2491 (31.3)
	Disagree	1210 (15.2)
	Agree	4269 (53.6)
*Primary predictors:*		
Fast food	Neutral	669 (8.4)
	Dislike	6276 (78.7)
	Like	234 (2.9)
	Do not eat	791 (9.9)
Salty snacks	Neutral	995 (12.5)
	Dislike	6202 (77.8)
	Like	386 (4.8)
	Do not eat	387 (4.9)
Fruits	Neutral	1400 (17.6)
	Dislike	509 (6.4)
	Like	6037 (75.7)
	Do not eat	24 (0.3)
Vegetables	Neutral	754 (9.5)
	Dislike	141 (1.8)
	Like	7060 (88.6)
	Do not eat	15 (0.2)
Sweetened beverages/soft drinks	Neutral	1993 (25.0)
	Dislike	4868 (61.1)
	Like	1012 (12.7)
	Do not eat	97 (1.2)
*Covariates:*		
Age, in years (mean ± SD)		60.1 ± 10.0
Gender	Male	803 (47.7)
	Female	4167 (52.3)
Disease status	No	6290 (78.9)
	Yes	1680 (21.1)
Employment status	No	4237 (53.2)
	Yes	3733 (46.8)
Marital Status	No	1121 (14.1)
	Yes	6849 (85.9)
Educational attainment	Lower education	3673 (46.1)
	Middle School/High school	3330 (41.8)
	Technical school	442 (5.5)
	University/college or above	525 (6.6)
Gross household income quintiles	1 (Lowest)	928 (11.6)
	2	4183 (52.5)
	3	2098 (26.3)
	4	617 (7.7)
	5 (Highest)	144 (1.8)
Community	Rural	4574 (57.4)
	Urban	3396 (42.6)
Province	North East	1284 (16.1)
	East Coastal	1470 (18.4)
	Central	1249 (15.7)
	South	2051 (25.7)
	Municipals	1916 (24.0)

**Table 2:** Prevalence rates and crude examinations of food preferences and psychological well-being measurements (*n* = 7970); Data: China Health and Nutrition Survey, 2011.

Psychological Well-Being	“I Had as Much Pep as I Had in 2010” (*n*, %)	“I Am as Happy Now as When I Was Younger” (*n*, %)	“As I Get Older, Things Are Better Than I Thought They Would Be” (*n*, %)
Measurements	Neutral	Neutral	Neutral
	Disagree	Disagree	Disagree
	Agree	Agree	Agree
**Fast food** (*p*-value)	<0.001	<0.001	<0.001
Neutral	242 (3.0)	277 (3.5)	269 (3.4)
	147 (1.8)	141 (1.8)	96 (1.2)
	280 (3.5)	251 (3.1)	304 (3.8)
Dislike	1621 (20.3)	1886 (23.7)	1935 (24.3)
	1841 (23.1)	1549 (19.4)	934 (11.7)
	2814 (35.3)	2841 (35.6)	3407 (42.7)
Like	36 (0.5)	53 (0.7)	51 (0.6)
	62 (0.8)	63 (0.8)	35 (0.4)
	136 (1.7)	118 (1.5)	148 (1.9)
Do not eat	187 (2.3)	244 (3.1)	236 (3.0)
	350 (4.4)	300 (3.8)	145 (1.8)
	254 (3.2)	247 (3.1)	410 (5.1)
**Salty snacks** (*p*-value)	<0.001	<0.001	<0.001
Neutral	329 (4.1)	377 (4.7)	373 (4.7)
	256 (3.2)	229 (2.9)	136 (1.7)
	410 (5.1)	389 (4.9)	486 (6.1)
Dislike	1602 (20.1)	1881 (23.6)	1914 (24.0)
	1849 (23.2)	1579 (19.8)	942 (11.8)
	2751 (34.5)	2742 (34.4)	3346 (42.0)
Like	64 (0.8)	81 (1.0)	90 (1.1)
	119 (1.5)	112 (1.4)	54 (0.7)
	203 (2.5)	193 (2.4)	242 (3.0)
Do not eat	91 (1.1)	121 (1.5)	114 (1.4)
	176 (2.2)	133 (1.7)	78 (1.0)
	120 (1.5)	133 (1.7)	195 (2.4)
**Fruits** (*p*-value)	<0.001	<0.001	<0.001
Neutral	487 (6.1)	572 (7.2)	566 (7.1)
	416 (5.2)	369 (4.6)	241 (3.0)
	497 (6.2)	459 (5.8)	593 (7.4)
Dislike	112 (1.4)	124 (1.6)	140 (1.8)
	201 (2.5)	159 (2.0)	96 (1.2)
	196 (2.5)	226 (2.8)	273 (3.4)
Like	1482 (18.6)	1757 (22.0)	1777 (22.3)
	1771 (22.2)	1518 (19.0)	865 (10.9)
	2784 (34.9)	2762 (34.7)	3395 (42.6)
Do not eat	5 (0.1)	7 (0.1)	8 (0.1)
	12 (0.2)	7 (0.1)	8 (0.1)
	7 (0.1)	10 (0.1)	8 (0.1)
**Vegetables** (*p*-value)	<0.001	<0.001	<0.001
Neutral	286 (3.6)	332 (4.2)	345 (4.3)
	255 (3.2)	223 (2.8)	143 (1.8)
	213 (2.7)	199 (2.5)	266 (3.3)
Dislike	34 (0.4)	43 (0.5)	46 (0.6)
	49 (0.6)	38 (0.5)	22 (0.3)
	58 (0.7)	60 (0.8)	73 (0.9)
Like	1760 (22.1)	2079 (26.1)	2094 (26.3)
	2090 (26.2)	1787 (22.4)	1041 (13.1)
	3210 (40.3)	3194 (40.1)	3925 (49.2)
Do not eat	6 (0.1)	6 (0.1)	6 (0.1)
	6 (0.1)	5 (0.1)	4 (0.1)
	3 (<0.1)	4 (0.1)	5 (0.1)
**Sweetened beverages** (*p*-value)	<0.001	<0.001	<0.001
Neutral	669 (8.4)	770 (9.7)	732 (9.2)
	551 (6.9)	515 (6.5)	304 (3.8)
	773 (9.7)	708 (8.9)	957 (12.0)
Dislike	1191 (14.9)	1393 (17.5)	1457 (18.3)
	1446 (18.1)	1212 (15.2)	716 (9.0)
	2231 (28.0)	2263 (28.4)	2695 (33.8)
Like	196 (2.5)	257 (3.2)	268 (3.4)
	367 (4.6)	298 (3.7)	171 (2.1)
	449 (5.6)	457 (5.7)	573 (7.2)
Do not eat	30 (0.4)	40 (0.5)	34 (0.4)
	36 (0.5)	28 (0.4)	19 (0.2)
	31 (0.4)	29 (0.4)	44 (0.6)

**Table 3:** Multinomial logistic regression model estimates of relationships between food preferences and psychological well-being, adjusted for covariates. Data: China Health and Nutrition Survey, 2011.

Multinomial Logistic Regression	Model 1	Model 2	Model 3
Psychological Well-Being	“I Had as Much as Pep as I Had in 2010”	“I Am as Happy Now as When I Was Younger”	“As I Get Older, Things Are Better Than I Thought They Would Be”
**Fast food**			
	Agree (β, SE), Disagree (β, SE)	Agree (β, SE), Disagree (β, SE)	Agree (β, SE), Disagree (β, SE)
Neutral	-	-	-
Dislike	(0.22, 0.12), (0.35, 0.14 *)	(0.35, 0.12 **), (0.26, 0.14)	(0.31, 0.11 *), (0.09, 0.16)
Like	(0.75, 0.22 **), (0.69, 0.25 *)	(0.45, 0.20), (0.54, 0.23 *)	(0.64, 0.20 **), (0.45, 0.27)
Do not eat	(0.24, 0.17), (0.66, 0.18 **)	(0.15, 0.16), (0.63, 0.17 **)	(0.44, 0.15 *), (0.10, 0.20)
**Salty snacks**			
	Agree (β, SE), Disagree (β, SE)	Agree (β, SE), Disagree (β, SE)	Agree (β, SE), Disagree (β, SE)
Neutral	-	-	-
Dislike	(0.09, 0.10), (0.10, 0.12)	(<0.01, 0.10), (0.13, 0.11)	(0.06, 0.10), (0.18, 0.14)
Like	(0.50, 0.18 *), (0.37, 0.19)	(0.44, 0.17 *), (0.45, 0.18 *)	(0.35, 0.15), (0.23, 0.22)
Do not eat	(0.14, 0.21), (0.18, 0.20)	(0.27, 0.20), (−0.02, 0.19)	(0.17, 0.18), (0.33, 0.23)
**Fruits**			
	Agree (β, SE), Disagree (β, SE)	Agree (β, SE), Disagree (β, SE)	Agree (β, SE), Disagree (β, SE)
Neutral	-	-	-
Dislike	(0.31, 0.14), (0.63, 0.15 *)	(0.61, 0.14 **), (0.65, 0.15 **)	(0.44, 0.13 **), (0.43, 0.17 *)
Like	(0.39, 0.08 **), (0.24, 0.09)	(0.45, 0.08 **), (0.22, 0.09 *)	(0.36, 0.08 **), (0.05, 0.10)
Do not eat	(1.99, 1.14), (2.17, 1.14)	(1.63, 0.75), (0.38, 0.84)	(0.14, 0.72), (0.87, 0.75)
**Vegetables**			
	Agree (β, SE), Disagree (β, SE)	Agree (β, SE), Disagree (β, SE)	Agree (β, SE), Disagree (β, SE)
Neutral	-	-	-
Dislike	(0.50, 0.25), (−0.10, 0.26)	(0.34, 0.24), (−0.23, 0.26)	(0.39, 0.22), (−0.23, 0.30)
Like	(0.53, 0.11 **), (0.09, 0.11)	(0.53, 0.11 **), (0.09, 0.11)	(0.59, 0.10 **), (0.15, 0.12)
Do not eat	(−1.60, 1.26), (−2.02, 1.24)	(−0.52, 0.95), (0.09, 0.97)	(0.19, 0.87), (−0.47, 0.97)
**Sweetened beverages**			
	Agree (β, SE), Disagree (β, SE)	Agree (β, SE), Disagree (β, SE)	Agree (β, SE), Disagree (β, SE)
Neutral	-	-	-
Dislike	(0.40, 0.07 **), (0.36, 0.08 **)	(0.45, 0.07 **), (0.24, 0.07 **)	(0.29, 0.06 **), (0.15, 0.09)
Like	(0.53, 0.10 **), (0.70, 0.11 **)	(0.54, 0.10 **), (0.38, 0.11 **)	(0.33, 0.09 **), (0.31, 0.12 *)
Do not eat	(0.19, 0.30), (−0.25, 0.30)	(−0.07, 0.29), (−0.41, 0.29)	(0.11, 0.27), (−0.13, 0.35)

* *p*-value < 0.05; ** *p*-value < 0.01 (adjusted for Benjamini-Hochberg’s false detection rate); Model 1: “I had as much pep as I had in 2010” = Age + Gender + Disease status + Employment status + Marital status + Educational attainment + Gross household income quintiles + Community + Province + Fast food + Salty snacks + Fruits + Vegetables + Sweetened beverages/Soft drinks; Model 2: “I am as happy now as when I was younger” = Age + Gender + Disease status + Employment status + Marital status + Educational attainment + Gross household income quintiles + Community + Province + Fast food + Salty snacks + Fruits + Vegetables + Sweetened beverages/Soft drinks; Model 3: “As I get older, things are better than I thought they would be” = Age + Gender + Disease status + Employment status + Marital status + Educational attainment + Gross household income quintiles + Community + Province + Fast food + Salty snacks + Fruits + Vegetables + Sweetened beverages/Soft drinks.

**Table 4:** Relative risk ratios (RRR) estimated by multinomial regression models on associations between five food preferences and psychological well-being measures adjusted with all covariates. Data: China Health and Nutrition Survey, 2011.

Multinomial Logistic Regression	Model 1	Model 2	Model 3
	RRR	RRR	RRR
	“I Had as Much as Pep as I Had in 2010”	“I Am as Happy Now as When I Was Younger”	“As I Get Older, Things Are Better Than I Thought They Would Be”
**Fast food**			
	Agree, Disagree	Agree, Disagree	Agree, Disagree
Neutral	-	-	-
Dislike	1.25, 1.41 *	1.43 **, 1.29	1.36 *, 1.10
Like	2.11 **, 2.00 *	1.57, 1.71 *	1.91 **, 1.56
Do not eat	1.28, 1.94 **	1.16, 1.87 **	1.55 *, 1.10
**Salty snacks**			
	Agree, Disagree	Agree, Disagree	Agree, Disagree
Neutral	-	-	-
Dislike	1.09, 1.10	1.00, 1.14	1.07, 1.20
Like	1.65 *, 1.45	1.55 *, 1.56 *	1.41, 1.25
Do not eat	1.15, 1.19	1.31, 0.98	1.18, 1.39
**Fruits**			
	Agree, Disagree	Agree, Disagree	Agree, Disagree
Neutral	-	-	-
Dislike	1.36, 1.87 *	1.85 **, 1.91 **	1.55 **, 1.53 *
Like	1.47 **, 1.27	1.57 **, 1.24 *	1.44 **, 1.05
Do not eat	7.32, 8.72	5.12, 1.47	1.15, 2.40
**Vegetables**			
	Agree, Disagree	Agree, Disagree	Agree, Disagree
Neutral	-	-	-
Dislike	1.65, 0.90	1.40, 0.79	1.48, 0.79
Like	1.70 **, 1.09	1.70 **, 1.10	1.81 **, 1.16
Do not eat	0.20, 0.13	0.59, 1.09	1.21, 0.63
**Sweetened beverages/Soft drinks**			
	Agree, Disagree	Agree, Disagree	Agree, Disagree
Neutral	-	-	-
Dislike	1.49 **, 1.43 **	1.56 **, 1.27 **	1.33 **, 1.16
Like	1.70 **, 2.01 **	1.72 **, 1.46 **	1.38 **, 1.36 *
Do not eat	1.20, 0.78	0.93, 0.66	1.11, 0.88

* *p*-value < 0.05; ** *p*-value < 0.01 (adjusted after Benjamini-Hochberg’s false detection rate); Reference level (Ref.) = 1.00; Model 1: “I had as much pep as I had in 2010” = Age + Gender + Disease status + Employment status + Marital status + Educational attainment + Gross household income quintiles + Community + Province + Fast food + Salty snacks + Fruits + Vegetables + Sweetened beverages/Soft drinks; Model 2: “I am as happy now as I was younger” = Age + Gender + Disease status + Employment status + Marital status + Educational attainment + Gross household income quintiles + Community + Province + Fast food + Salty snacks + Fruits + Vegetables + Sweetened beverages/Soft drinks; Model 3: “As I get older, things are better than I thought they would be” = Age + Gender + Disease status + Employment status + Marital status + Educational attainment + Gross household income quintiles + Community + Province + Fast food + Salty snacks + Fruits + Vegetables + Sweetened beverages/Soft drinks.
